# Ascites—II

**Published:** 1909-06-19

**Authors:** 


### Medicine.

### ASCITES—II.

### PHYSICAL SIGNS (*continued*).

By far the most important sign of ascites is the effect which is produced on the line of dulness by a change in the posture of the patient. If there is dulness in the flanks and resonance over the front of the abdomen when the patient is lying perfectly flat upon his back, it will be found that if he turns and lies on one side the uppermost flank becomes resonant and the line of dulness on the other side rises towards the median abdominal line. This phenomenon is clearly due to the fluid gravitating to the most dependent parts. It is very helpful sometimes in distinguishing between an impaired note in the flank due to free ascitic or peritonitic fluid on the one hand, or to absence of air from the colon owing to its collapse or to its being overloaded with fæces on the other.

It sometimes happens that owing to adhesions and the matting together of the intestines in cases of chronic peritonitis, the ascitic fluid becomes shut off as local collections, in which case the phenomenon of gravitation to the lowest part of the abdominal cavity no longer occurs. The diagnosis of such cases of "encysted ascites" must clearly be difficult unless the case has been followed from a time at which the ascitic fluid was not yet shut off by adhesions in this way.

In ordinary cases of ascites the liver is apt to be pushed upwards, and in consequence the right side of the chest becomes dull to percussion at the fifth or fourth or even the third intercostal space in the nipple line instead of at the sixth as usual.

If only a very small quantity of fluid is present the abdomen may be resonant all over when the patient lies on his back; if such be the case, and the posture be now changed for that of the knee-elbow position, the umbilical region—previously resonant—will be found to have become dull.

Auscultation is seldom of any assistance in the diagnosis of ascites. The most that can be said of it is that now and then in a case of ascites due to inflammation of the peritoneum a friction rub can be detected over the liver or the spleen when the patient takes a particularly deep breath.

Mensuration may be of assistance in making the differential diagnosis of ascites. In a typical case the maximum circumference of the abdomen is at the level of the umbilicus. The latter remains nearer to the pubes than to the ensiform cartilage just as it normally is, and it is equidistant from each anterior superior iliac spine.

In women it may be necessary to make a vaginal examination. The vagina is usually felt to be shortened; the uterus may still be freely movable, but pushed downwards and flexed; and Douglas's pouch may be obviously distended with fluid.

Now and then it is not until a trochar and cannula have been used to withdraw some of the fluid that the cause of the abdominal distension can be determined. In ascites the fluid varies greatly according as it has arisen by transudation or by exudation. If due to transudation it is usually clear, pale yellow, alkaline, and of specific gravity, 1,012 or less; it does not coagulate spontaneously on standing; it contains a small amount of total solids—from 1.5 to 2 per cent.; it is free from peptone, but contains serum-albumen; microscopically it is found to contain few cell elements, though in exceptional cases the fluid has been known to be hæmorrhagic or chylous. If due to exudation, the fluid may be clear, but more often is slightly turbid and yellowish or greenish-yellow; it is alkaline, and the specific gravity is usually over 1,015; the exudate may form a thin, floating clot on standing, usually within twenty-four hours, and often in a much shorter time than this. Serum albumen, serum globulin, and traces of uric acid, urea, and sugar are present. Microscopically numerous cell elements are seen, a few red blood-corpuscles, leucocytes, large peritoneal squamous cells, and granular detritus. Exudations may also be hæmorrhagic, sero-purulent, purulent, or chylous.

### The Diagnosis of Ascites from Other Conditions.

Amongst conditions that may lead to a diagnosis of ascites when none is present may be mentioned: (1) Tympanites, and the various causes of it; (2) ovarian cyst; (3) pregnancy; (4) obesity; (5) an enormously distended bladder; (6) phantom tumour; (7) hydronephrosis; (8) pancreatic cyst; (9) enormous enlargement of liver or spleen; (10) enormous dilatation of the stomach. A brief discussion of the main points of distinction in each of the above becomes necessary.

Tympanites is the term used to signify distension of the abdomen from excessive accumulation of gas within the intestine, or more rarely from the presence of free gas in the peritoneal cavity. The following are some of the causes of tympanites: Intestinal obstruction; bowel paralysis from acute peritonitis; certain acute diseases, such as typhoid fever, lobar pneumonia, septicæmia, and so forth; perforation of the stomach or intestine from ulceration or from injury; myelitis, fractured spine, and some other affections of the spinal cord; hysteria.

Tympanites is distinguished by the following physical signs:—

The abdomen is uniformly distended, frequently to an enormous extent. It becomes globular in shape. There is no marked bulging of the flanks, and change of posture does not alter the shape as in ascites. The distension of the abdomen may be asymmetrical if the tympanites is due to obstruction of the upper part of the small intestine, or if any particular portion of the intestine becomes excessively distended. The outlines of distended coils of intestines may be visible, and if there is no peritonitis peristaltic movements may be observed. The skin is stretched, and it may be shiny. The umbilicus may or may not be raised level with the surrounding skin; its normal relative position is retained. The cardiac impulse may be displaced upwards and outwards.

The surface of the abdomen feels smooth, tense, and drum-like. In cases of chronic obstruction of the large intestine the distended coils of bowel may be felt, and sometimes the peristaltic movements in them. No fluid thrill can be made out.

There is a tympanitic note all over the abdomen, no matter in what position the patient is placed. The splenic dulness may be replaced by resonance. The liver dulness may be diminished or, in extreme cases, absent. It is of great moment to remember that diminution or absence of the hepatic dulness is often due to resonance from the distended colon being conveyed through the liver, and that therefore the sign is very far from signifying perforation of a hollow viscus as is sometimes stated.

(*To be continued.*)

